# Neurotrophic factor-α1/carboxypeptidase E regulates critical protein networks to rescue neurodegeneration, defective synaptogenesis and impaired autophagy in Alzheimer’s disease mice

**DOI:** 10.1186/s40035-025-00520-6

**Published:** 2025-11-26

**Authors:** Lan Xiao, Pranav Sharma, Xuyu Yang, Daniel Abebe, Y. Peng Loh

**Affiliations:** 1 Section on Cellular Neurobiology, Eunice Kennedy Shriver National Institute of Child Health and Human Development, National Institutes of Health, 49, Convent Drive, Bldg. 49, Rm 6A-10, NICHD, NIH, Bethesda, MD 20892 USA; 2 Xosomix LLC, 3210 Merryfield Row, San Diego, CA 92121 USA

**Keywords:** Alzheimer’s disease, Neurotrophic Factor-α1/carboxypeptidase E (NF-α1/CPE), Gene therapy, 3 × Tg AD, Autophagy, Synaptogenesis, Neurodegeneration

## Abstract

**Background:**

The global aging population is increasingly inflicted with Alzheimer’s disease (AD), but a cure is still unavailable. Neurotrophic factor-α1/carboxypeptidase E (NF-α1/CPE) gene therapy has been shown to prevent and reverse memory loss and pathology in AD mouse models. However, the mechanisms of action of NF-α1/CPE are not fully understood. We investigated if a non-enzymatic form of NF-α1/CPE-E342Q is efficient in reversing AD pathology and carried out a proteomic study to uncover the mechanisms of action of NF-α1/CPE in AD mice.

**Methods:**

AAV-human NF-α1/CPE or a non-enzymatic form, NF-α1/CPE-E342Q, was delivered into the hippocampus of 3 × Tg-AD male mice. The effects on cognitive function, neurodegeneration, synaptogenesis and autophagy were investigated. A quantitative proteomic analysis of the hippocampus was carried out.

**Results:**

Hippocampal delivery of AAV-NF-α1/CPE-E342Q prevented memory loss, neurodegeneration and microglial activation in 3 × Tg-AD mice, indicating that the action is independent of its enzymatic activity. Quantitative proteomic analysis of the hippocampus of 3 × Tg-AD mice revealed differential expression of > 2000 proteins involving many metabolic pathways after NF-α1/CPE gene therapy. Of these, two new proteins, Snx4 and Trim28, which increase Aβ production and tau levels, respectively, were down-regulated by NF-α1/CPE. Western blot analysis verified their reduction in AAV-NF-α1/CPE-treated 3 × Tg-AD mice compared to untreated mice. Our proteomic analysis indicated synaptic organization as the top signaling pathway altered in response to CPE expression. Synaptic markers PSD95 and Synapsin1 were decreased in 3 × Tg-AD mice and were restored with AAV-NF-α1/CPE treatment. Proteomic analysis hypothesized involvement of autophagic signaling pathway. Indeed, multiple protein markers of autophagy were down-regulated in 3 × Tg-AD mice, accounting for impaired autophagy. NF-α1/CPE gene therapy upregulated the levels of these proteins in 3 × Tg-AD mice, thereby reversing autophagic impairment.

**Conclusions:**

This study uncovered vast actions of NF-α1/CPE in restoring expression of networks of critical proteins including those necessary for maintaining neuronal survival, synaptogenesis and autophagy, while down-regulating many proteins that promote tau and Aβ accumulation to reverse memory loss and AD pathology in 3 × Tg-AD mice. AAV-NF-α1/CPE gene therapy uniquely targets many metabolic levels, offering a promising holistic approach for AD treatment (Graphical Abstract).

**Graphical abstract:**

**Figure:**
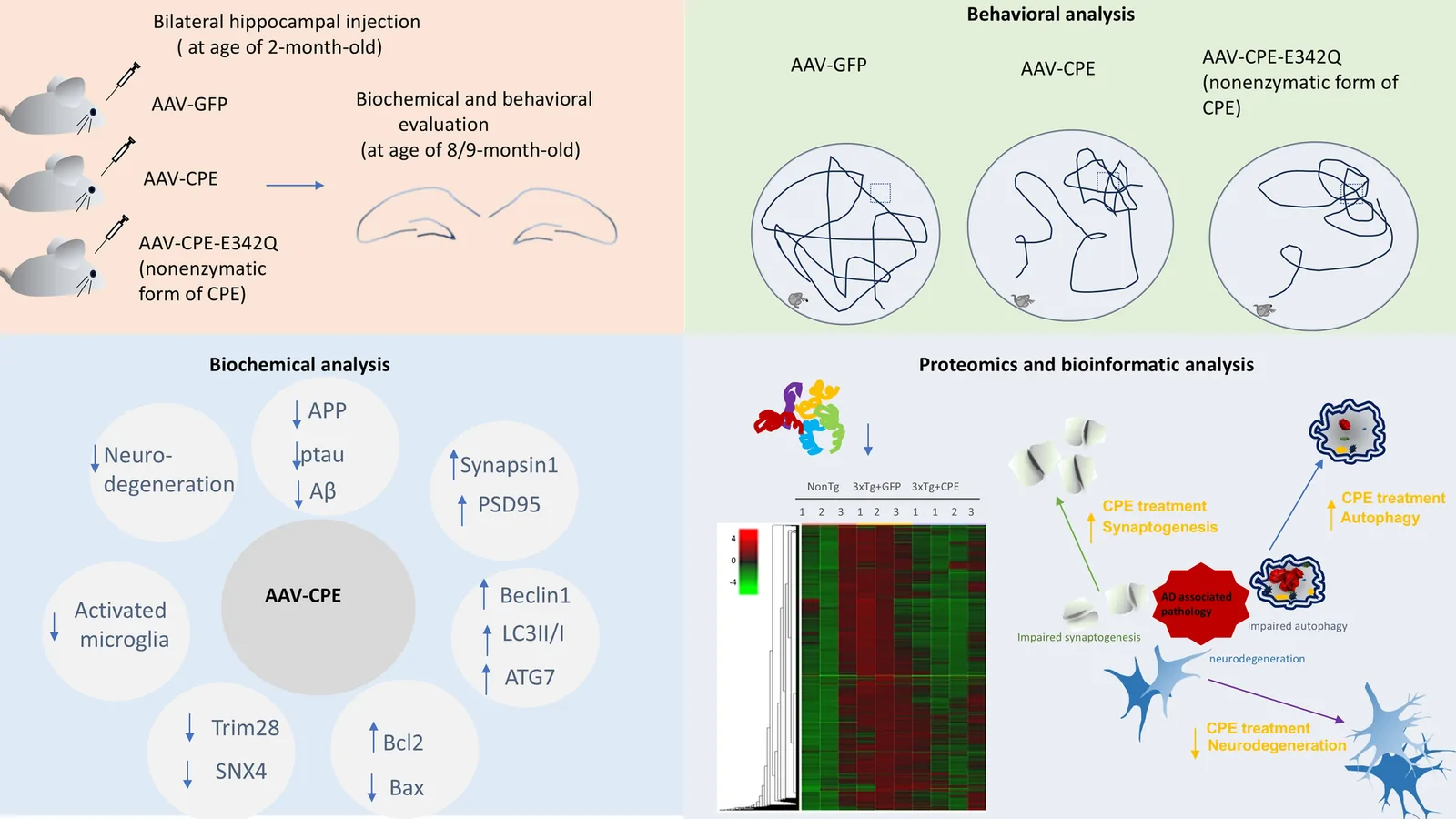

**Supplementary Information:**

The online version contains supplementary material available at 10.1186/s40035-025-00520-6.

## Introduction

Alzheimer’s disease (AD) is one of the most prevalent neurological disorders that lead to dementia worldwide. With an increasing ageing population globally, the number of patients diagnosed with AD is expected to reach 152 million by 2050 [1]. Both environmental and genetic components contribute to the pathogenesis of AD. A majority of patients demonstrate late-onset symptoms after age of 65, while approximately 5% of AD patients develop early-onset symptoms before age 65 due to genetic predisposition such as genetic mutations in amyloid precursor protein (*APP*) and presenilins 1/2 (*PSEN*1/*PSEN*2) [2]. Most AD patients demonstrate progressive memory loss, and impairment in visuospatial and executive function [3]. Pathologically, AD is characterized by severe neurodegeneration, abnormal extracellular aggregation of β-amyloid (Aβ) and intracellular aggregation of hyperphosphorylated tau in the central nervous system. Alterations of a variety of cellular activities are also closely related with the pathogenesis of AD. Autophagy plays a critical role in maintaining intracellular protein homeostasis. In the major autophagy–lysosome pathway, misfolded proteins or damaged organelles are encapsulated within autophagosomes and then transported to lysosomes for degradation. A large number of studies have reported that autophagy dysfunction is highly associated with accumulation of amyloid proteins and neurodegeneration [4]. In the brains of AD patients and PS1/APP mice, autophagosomes accumulate in the dystrophic neurites and contribute to the aggregation of Aβ [5]. On the other hand, reductions of synapses and synaptogenesis are also correlated with declined cognition and exacerbation of AD [6, 7]. Specially, pathological tau is linked with synaptic loss and synaptic dysfunction [8]. Although AD has been known for more than a hundred years since first description in 1906 [9, 10], the regulation of the deficits remains poorly understood.

Neurotrophic factor-α1/carboxypeptidase E (NF-α1/CPE), which exerts neuroprotective effects in both in vitro and in vivo studies [11, 12], has recently been demonstrated to prevent and reverse memory loss and AD pathology in a mouse model [13]. CPE-KO mice display hippocampal CA3 neurodegeneration and impaired learning and memory [14]. Mice carrying a mutated *CPE* gene also demonstrate abnormal hippocampal function and deficits in cognition [14, 15]. In humans, associations between *CPE* mutations and cognitive impairment and learning disability have been reported in several clinical cases [16, 17]. Interestingly, studies have indicated that although CPE plays a critical role in processing proneuropeptide, its neuroprotective activity is independent of its enzymatic activity. This is supported by studies showing that transgenic mice carrying the non-enzymatic CPE-E342Q display intact hippocampal structure and normal memory and learning, in comparison with CPE-KO mice[12].

Studies in human neurons support the involvement of CPE in neuroprotection. CPE is mainly distributed in neurons, astrocytes and glia in the cortex of humans. In the cortex of AD patients, CPE accumulates in dystrophic neurites around amyloid plaques [18]. Another study showed that NF-α1/CPE is co-localized with the G-protein-coupled serotonin receptor HTR1E in human hippocampal neurons. Secreted NF-α1/CPE interacts with HTR1E, recruiting β-arrestin and activating the ERK-CREB signaling pathway, resulting in upregulation of Bcl2 signaling and enhancement of neuronal survival [19]. Although the receptor has not yet been identified in mice, studies have shown that the NF-α1/CPE-mediated neuroprotection in mice also involves activation of the ERK-Bcl2 signaling pathway [12].

Our previous studies have shown that hippocampal delivery of AAV-NF-α1/CPE (mouse) in 3 × Tg-AD mouse model prevented cognitive decline, neurodegeneration, and amyloid and tau pathology [13]. To further elucidate the mechanisms by which NF-α1/CPE rescues AD pathology, in this study, we plan to inject AAV-human NF-α1/CPE or a non-enzymatic form of human NF-α1/CPE (E342Q) in the hippocampus of 3 × Tg-AD male mice and carry out an extensive proteomic analysis to identify critical proteins that are modulated by NF-α1/CPE to rescue cognitive dysfunction. The quantitative proteomic analysis will also provide us with protein signaling networks that result from the expression of CPE gene in 3 × Tg-AD versus Tg-AD mice. Such data will generate testable hypotheses that would further help investigations into the mechanisms used by CPE to rescue deficits in the brains of 3 × Tg-AD mice.

## Materials and methods

### Animals

The mouse strain used for this research project, B6;129-Tg(APPSwe,tauP301L)1Lfa *Psen1*^*tm1Mpm*^/Mmjax (RRID:MMRRC_034830-JAX), was obtained from the Mutant Mouse Resource and Research Center (MMRRC) at The Jackson Laboratory, an NIH-funded strain repository, and was donated to the MMRRC by Frank Laferla, Ph.D., University of California, Irvine; Mark P. Mattson, Ph.D., Johns Hopkins University, School of Medicine [20–23]. Male mice were used in this study. All mice were housed at NIH animal facility with free access to food and water at controlled humidity (45%) and temperature (22 °C) under a 12-h light/dark cycle. At age of ~ 2 months, 3 × Tg-AD and nonTg mice were randomly selected and divided into 4 groups: nonTg + GFP, 3 × Tg + GFP, 3 × Tg + NF-α1/CPE and 3 × Tg + NF-α1/CPE-E342Q to receive bilateral hippocampal stereotaxic injections. At age of ~ 8 months, brain tissues were collected for biochemical and immunohistochemical studies after behavioral tests.

### Viral vectors

AAV1/2-GFP and AAV1/2-CPE (chimeric serotype) viral constructs were purchased from Vector Biolabs (Philadelphia, PA). AAV1 and AAV2 transduce neurons, reactive microglia and astrocytes [24]. GFP and CPE expressions in these AAV constructs were driven by the CMV promoter.

### Stereotaxic injection

Stereotaxic injection was conducted as previously described [13]. AAV viruses expressing GFP or human NF-α1/CPE or NF-α1/CPE-E342Q were bilaterally injected into the hippocampus (total 1 × 10^10^ VP, 1 μL on each side of hippocampus) according to the following coordinates: AP, − 1.94 mm, L: ± 1.0 mm, V: − 1.3 mm.

### Electronic microscopy (EM)

For transcardial perfusion fixation, the mice were deeply anesthetized under 2.5% in 2 litter/min oxygen (V/V) isoflurane using a VetEquip Vaporizer (VetEquip Inc., Marsing, ID) until a toe pinch yields no response. Then the mice were transcardially perfused with 4% paraformaldehyde (PFA) with 2.5% glutaraldehyde in PBS buffer, pH 7.4. Brains were removed and left to post-fix overnight in the same fixative at 4 °C. After primary fixation and dissection, samples were cut at 100 μm on a Vibratome (LEICA VT1000, Leica Biosystems, S. Deer Park, IL). Next, regions of interest were selected, cut and inserted into mPrep tissue capsules and loaded onto an mPrep ASP-2000 Automated Biological Specimen Preparation Processor (Microscopy Innovations LLC, Marshfield, WI) which automates all processing steps, including post-fixation in 2% osmium tetroxide, en-bloc in 2% uranyl acetate (aqueous), dehydration in a graded ethanol series followed by further dehydration in 100% acetone, and finally infiltrated and embedded in Embed 812 epoxy resin (Electron Microscopy Sciences, Hatfield, PA). Embedded samples were polymerized in an oven set at 60 °C. Samples were then ultra-thin sectioned (90 nm) on a Leica EM UC7 Ultramicrotome. Thin sections were picked up and placed on 200-mesh copper grids and post-stained with UranyLess (Uranyl Acetate substitute, Electron Microscopy Sciences) and lead citrate. Imaging was performed on a JEOL-1400 Transmission Electron Microscope (Peabody, MA**)** operating at 80 kV with an AMT BioSprint-29 camera (Advanced MicroscopyTechniques, Woburn, MA).

### Behavioral studies

Male nonTg and 3 × Tg-AD mice at 2 months of age were injected with AAV-GFP, AAV-NF-α1/CPE or NF-α1/CPE-E342Q in the hippocampus and then tested for open field and Morris water maze at ~ 8 months of age.

#### Open field test

To evaluate the locomotor activity, each mouse was tested in an open field apparatus for 1 h, and the distance and the speed of traveling were evaluated by the ANY-maze system (ANY-maze, Wood Dale, IL).

#### Morris water maze

Morris water maze was conducted as previously described [25], and consisted of two sessions: a hidden-platform training session (on days 1–5) and a probe test session (on day 6). The test was performed in a circular pool full of water and nontoxic white paint. On days 1–5, the hidden platform was positioned in the same spot and mice were allowed to search for the platform for 1 min. There were four trials each day and mice were placed in a new quadrant of the pool in each trial. If mice failed to find the hidden platform, they were guided to the platform and allowed to sit on it for 30 s. On day 6, the hidden platform was removed, and mice were allowed to explore the pool for 1 min. The time in each quadrant was recorded and analyzed by the ANY-maze system.

### Aβ40 and Aβ42 ELISA

Mouse brain tissues were processed as previously described [26]. Hippocampal tissues were homogenized and centrifuged for 30 min at 100,000 × *g* at 4 °C. The supernatant which contains soluble proteins was collected. The pellet which contains insoluble proteins was resuspended in 70% formic acid (Sigma-Aldrich, St. Louis, MO). The supernatant and the pellet were analyzed for soluble and insoluble Aβ40 and Aβ42 using ELISA kits (Cat # KHB3481, Cat # KHB3441, Invitrogen, Waltham, MA) following the manufacture’s protocol.

### Western blot

Mouse brain tissues were prepared as previously described [25]. Hippocampal lysates were run on SDS-PAGE gel and transferred onto nitrocellulose membranes. The membranes were then incubated with primary antibodies (Table S1) overnight followed by secondary fluorescent conjugated anti-mouse or -rabbit antibodies (Cat # 926–66072; Cat # 926–32211, Licor Inc, Lincoln, NE). The proteins were visualized and protein level quantified by the Odyssey infrared imaging system (LI-COR Inc, Lincoln, NE) and normalized to β-actin protein level.

### Immunohistochemistry

Mouse brains were sectioned coronally at 25 µm and then incubated with primary antibodies (Table S2) and then with biotinylated (1:1000, Cat # ba-1000, Vector, Newark, CA) or fluorescence (1:500, Cat # 711-165-152, Cat # 703-165-155; Jackson Lab, Bar Harbor, ME) secondary antibodies. Images were scanned with an Olympus VS200 slide scanning system. For MAP2 and GFAP quantification, two random areas in CA1 region (167 μm × 167 μm) (four sections per animal, six animals per genotype) were selected. MAP2 intensity was measured by Image J and GFAP-positive cells were counted. To quantify CD68-positive cells, the numbers of positive cells and total cells in CA1 region were counted within an area (~ 160 μm × 320 μm) (four sections per mouse, 6 mice per genotype). The percentage of positive cells to total cells was calculated.

### Proteomic analysis

#### Cell lysate preparation

Hippocampal tissues were dissected from 3 groups of mice: nonTg + GFP, 3 × Tg + GFP, and 3 × Tg + CPE in triplicates. Cell lysates were prepared in RIPA buffer (Thermo Fisher Scientific, Waltham, MA) added with Halt™ Protease and Phosphatase Inhibitor Cocktail (Thermo Fisher Scientific).

#### Protein digestion protocol

Protein concentrations were determined using the micro-BCA Protein Assay Kit (Thermo Fisher Scientific) for each of the 9 cell lysates. Equal amounts of protein for each group were precipitated using ammonium sulphate. The pellets were resuspended in 600 μL of 8 mol/L urea in 100 mmol/L Tris pH 8.0 by vortexing for 5–10 min. Tris(2-carboxyethyl)phosphine hydrochloride was added to a final concentration of 10 mmol/L. Samples were then frozen overnight at − 20 °C for solubilization of the proteins in urea solution. Next day, the solution was thawed and vortexed for another 5 min until the solution became clear. The chloro-acetamide solution was added to a final concentration of 40 mmol/L and vortexed for 5 min. Equal volumes of 50 mmol/L Tris pH 8.0 was added to the sample to reduce the urea concentration to 4 mol/L. Lys C was added at a 1:500 ratio to protein content and incubated at 37 °C in a rotating incubator for 4–6 h. Then equal volumes of 50 mmol/L Tris pH 8.0 was added to the sample to reduce the urea concentration to 2 mol/L. Trypsin was added at a 1:50 ratio and incubated overnight at 37 °C. The solution was then acidified using trifluoroacetic acid (TFA, 0.5% TFA final concentration) and vortexed for 5 min followed by centrifugation at 14,000 × *g* for 5 min to obtain aqueous and organic phases. The lower aqueous phase was collected and desalted using 100 mg C18-StageTips as described in the manufacturer’s protocol. The peptide concentration in the sample was measured using BCA after resuspension in iTRAQ dissolution buffer.

#### Tandem mass tags (TMT) labeling

TMT tags from Thermo Scientific TMT10plex** (**Cat # 90110) were used for the labeling, according to the manufacturer’s protocol.

#### High pH fractionation

Pierce™ High pH Reversed-Phase Peptide Fractionation Kit (Cat #84868) was used to fractionate labeled peptides according to the manufacturer’s protocol.

#### Liquid chromatography coupled with tandem mass spectroscopy (LC–MS/MS)

Each fraction was analyzed by ultra-high-pressure liquid chromatography (UPLC) coupled with tandem mass spectroscopy using nano-spray ionization. The nano-spray ionization experiments were performed using an Orbitrap fusion Lumos hybrid mass spectrometer (Thermo) interfaced with nanoscale reversed-phase UPLC (Thermo Dionex UltiMate™ 3000 RSLC nano System) using a 25 cm, 75-micron ID glass capillary packed with 1.7-µm C18 (130) BEH™ beads (Waters Corporation, Milford, MA). Peptides were eluted from the C18 column into the mass spectrometer, using a linear gradient (5%–80%) of acetonitrile (ACN) at a flow rate of 375 μL/min for 180 min. The buffers used to create the ACN gradient were Buffer A (98% H_2_O, 2% ACN, 0.1% formic acid) and Buffer B (100% ACN, 0.1% formic acid). Mass spectrometer parameters are as follows: an MS1 survey scan using the Orbitrap Detector (mass range: 400–1500 m/z (using quadrupole isolation), 60,000 resolution setting, spray voltage: 2200 V, ion transfer tube temperature: 275 °C, AGC target: 400,000, and maximum injection time: 50 ms), followed by data-dependent scans (top speed for most-intense ions, with charge state set to only include + 2–5 ions, and 5-s exclusion time), while selecting ions with minimal intensities of 50,000 for collision in the high energy collision cell (HCD Collision Energy, 38%) and the first quadrupole isolation window was set at 0.7 (m/z). The fragment masses were analyzed in the Orbi-trap mass analyzer with a mass resolution setting of 15,000 (ion trap scan rate: turbo, first mass m/z: 100, AGC Target: 20,000 and maximum injection time: 22 ms). Protein identification and quantification were carried out using the Peaks Studio X software (Bioinformatics Solutions Inc, Waterloo, Ontario, Canada).

#### Differential expression analysis

Protein intensities were normalized by applying log2 transformation to reduce skewness and median normalization to account for variations in sample loading and global batch intensity differences. Principal component analysis (PCA) implemented with the R package *prcomp*, was used to evaluate the influence of covariates such as batch, sex, age, and genotype, ensuring that the biological signals were not confounded by confounding variables. Differential expression (DE) analysis was performed using a moderated *t*-test from the *MKmisc* package, which estimates the variance by borrowing information across all proteins, providing increased statistical power compared to traditional *t*-tests. To control for false discoveries in multiple testing, the *P* values were adjusted to false discovery rate (FDR) using the Benjamini–Hochberg procedure.

#### Pathway enrichment and protein–protein interaction network analysis

Pathway enrichment of differentially expressed proteins (DEPs) was performed using the STRING software. Pathway enrichment specifically emphasized the Gene Ontology (GO) categories relevant to neurodegenerative processes, informed by prior knowledge of AD pathogenesis. Results were filtered by FDR < 0.01 to identify DEP-associated pathways with high confidence. PANTHER Overrepresentation Test [27] was used to assess whether specific gene sets are represented in the input gene list differently from what is expected by chance. Positive values represent fold over-representation while negative value represents fold under-representation. We used Fisher’s exact test with FDR to calculate *P*-values. A significant *P*-value indicates over-representation or under-representation of a gene set for a specific function.

### Statistical analysis

Data are representative of at least 3 separate experiments (*N*), and each experiment was done in triplicates (*n* = 3) unless specified otherwise. Data were analyzed by two-tail Student’s *t*-test, or one-way ANOVA or two-way ANOVA followed by Tukey’s post-hoc multiple comparisons tests, where noted. Analysis was performed using the GraphPad Prism software package (GraphPad, La Jolla, CA). Significance was set at *P* < 0.05.

## Results

### Hippocampal delivery of AAV-NF-α1/CPE and AAV-NF-α1/CPE-E342Q rescues memory deficits in 3 × Tg-AD male mice

**Fig. 1:**
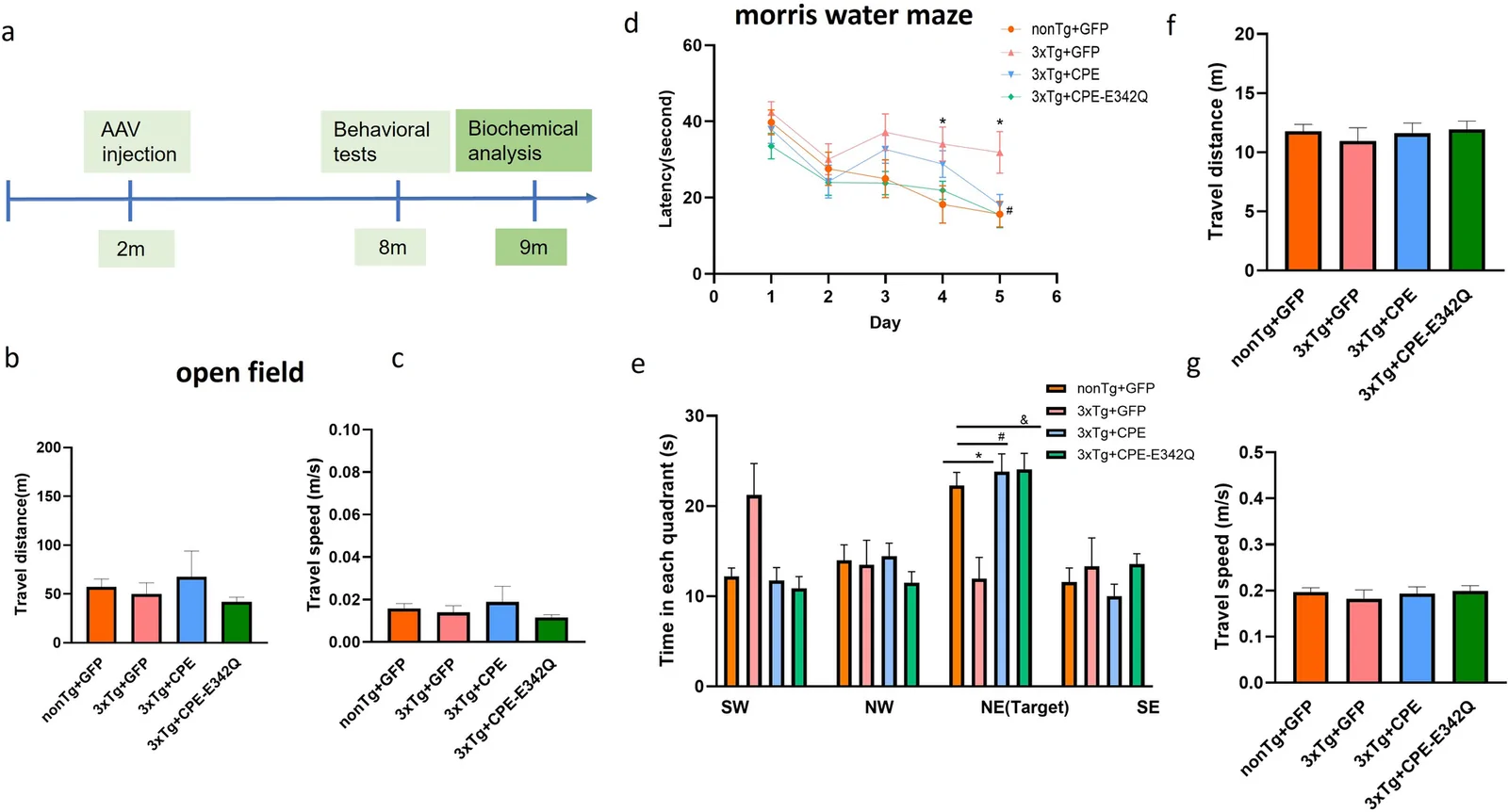
AAV-NF-α1/CPE and AAV-NF-α1/CPE-E342Q rescue memory deficits in 3 × Tg -AD mice. **a** Schematic graph of the experimental design. **b**, **c** Locomotor activity in the open field test. There were no significant differences across the groups in the travel distance and speed in the open field test. **d** Effects of overexpression of NF-α1/CPE or NF-α1/CPE-E342Q on spatial learning in the Morris water maze test. On day 4, the latency in the 3 × Tg + GFP group was increased compared to the nonTg + GFP (^*^P = 0.0363). One-way ANOVA analysis followed by Tukey’s post-hoc multiple comparison test [*F*(3,38) = 3.221, *P* = 0.0332]. On day 5, the latency in the 3 × Tg + GFP group was increased compared to nonTg + GFP (^*^P = 0.0284). NF-α1/CPE-E342Q treatment significantly reduced the latency in 3 × Tg-AD mice compared to 3 × Tg + GFP (^#^*P* = 0.0224), while NF-α1/CPE approached significance in reducing the latency (*P* = 0.0682). One-way ANOVA followed by Tukey’s post-hoc multiple comparison test [*F*(3,38) = 4.033, *P* = 0.0139]. **e** Overexpression of NF-α1/CPE or NF-α1/CPE-E342Q prevented memory deficits of 3 × Tg-AD in the Morris water maze. 3 × Tg + GFP mice spent less time in the target area (NE) compared to nonTg + GFP (^*^P = 0.0232), while both 3 × Tg + NF-α1/CPE (^#^*P* = 0.0022) and 3 × Tg + NF-α1/CPE-E342Q (^&^*P* = 0.0015) spent more time in the target quadrant compared to 3 × Tg + GFP. One-way ANOVA followed by Tukey’s post-hoc multiple comparison test [*F*(15,152) = 6.566, *P* < 0.0001]. *n* = 10–11, mean ± SEM. **f, g** There were no significant differences in swimming distance or speed across all groups in the Morris water maze. *n* = 10–11, mean ± SEM

3 × Tg-AD male mice received bilateral hippocampal injections of AAV-GFP, NF-α1/CPE, or NF-α1/CPE-E342Q at age of 2 months (presymptomatic). Non-Tg male mice received AAV-GFP injection. All mice underwent a series of behavioral tests at age of 8 months when cognitive decline was evident (Fig. 1a). Mice were first evaluated for locomotor activity in the open field test. There were no significant differences across all four groups in the traveling distance or speed (Fig. 1b, c). In the Morris water maze, the NF-α1/CPE-E342Q-treated mice exhibited significantly decreased latency on day 5 of training (Fig. 1d), and spent significantly more time in the target quadrant compared to the 3 × Tg + GFP mice in the probe test (Fig. 1e). All four groups of mice did not show any significant difference in the swimming speed or distance in the probe test (Fig. 1f, g).

### AAV-NF-α1/CPE and AAV-NF-α1/CPE-E342Q treatment rescues hippocampal CA1 neurodegeneration and decreases activated microglia in 3 × Tg-AD mice

**Fig. 2:**
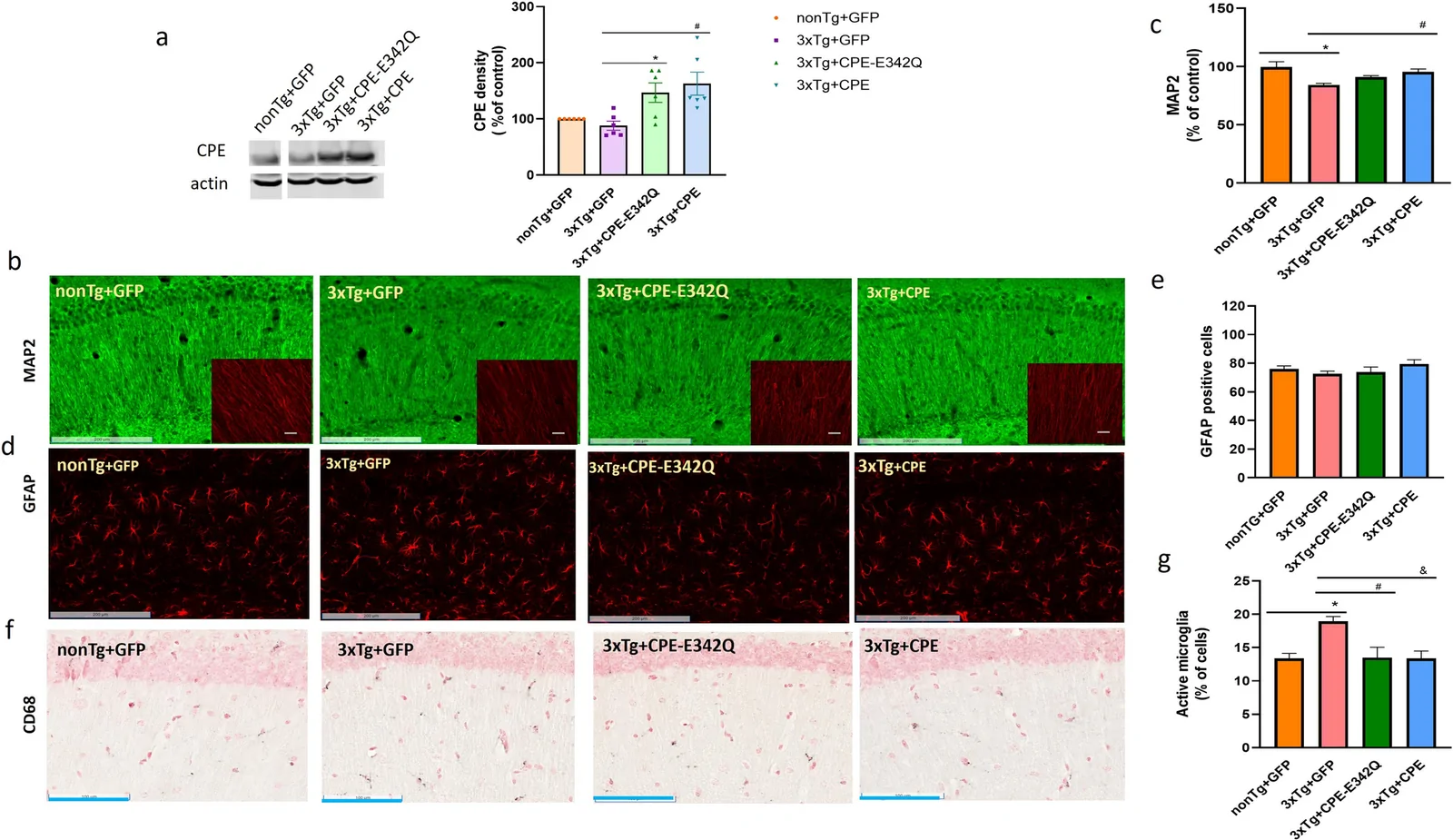
AAV-NF-α1/CPE and AAV-NF-α1/CPE-E342Q treatment rescues hippocampal CA1 neurodegeneration and decreases activated microglia. **a** CPE protein level was increased in the hippocampus of 3 × Tg-AD that received AAV-NF-α1/CPE (^#^*P* = 0.0059) or AAV-NF-α1/CPE-E342Q (^*^P = 0.0351) compared with 3 × Tg + GFP. One-way ANOVA analysis followed by Tukey’s post-hoc multiple comparison test [*F*(3,20) = 6.643, *P* < 0.01]. *n* = 6, mean ± SEM. **b, d, f** Representative immunohistochemistry images of MAP2 (**b**), GFAP (**d**) and CD68 (**f**) in the hippocampal CA1 region of nonTg + GFP, 3 × Tg + GFP, 3 × Tg + CPE-E342Q and 3 × Tg + CPE mice at the age of 8–9 months. Scale bars, 200 μm for **b** and **d**, 100 μm for** f**, 25 μm for red insets which were color converted from green in **b**. *n* = 6 mice per genotype. **c** Quantification of MAP2 intensity in hippocampal CA1 region at the age of 8–9 months. MAP2 intensity decreased in 3 × Tg + GFP compared with nonTg + GFP (^*^P = 0.0024); 3 × Tg + CPE increased MAP2 intensity significantly compared with 3 × Tg + GFP (^#^*P* = 0.0284). One-way ANOVA followed by Tukey’s post-hoc multiple comparison test [*F*(3,17) = 6.871, *P* = 0.0031]. mean ± SEM. 3–4 sections per mouse, *n* = 5–6 mice per genotype. **e** Quantification of GFAP-positive cells in hippocampal CA1 region at the age of 8–9 months. There were no significant changes across groups. One-way ANOVA followed by Tukey’s post-hoc multiple comparison test. *n* = 5–6 mice per genotype, 4 sections per mouse; mean ± SEM. **g** Quantification of CD68-positive cells in hippocampal CA1 at the age of 8–9 months. CD68-positive cells were significantly increased in 3 × Tg + GFP compared with nonTg + GFP (^*^P = 0.0141). Overexpression of NF-α1/CPE-E342Q or NF-α1/CPE in 3 × Tg-AD significantly reduced activated microglia (CPE-E343Q, ^#^*P* = 0.0119; CPE: ^&^*P* = 0.0142). One-way ANOVA followed by Tukey’s post-hoc multiple comparison test [*F*(3,18) = 6.196, *P* = 0.0044]. *n* = 5–6 mice per genotype, 4 sections per mouse; mean ± SEM

Western blotting showed that the protein levels of NF-α1/CPE and NF-α1/CPE-E342Q were significantly increased in 3 × Tg-AD mice after AAV-NF-α1/CPE and AAV-NF-α1/CPE-E342Q treatment, compared to those treated with GFP (Fig. 2a). Hippocampal CA1 neurodegeneration is significantly associated with AD [28]. MAP2 immunostaining in the CA1 region showed neurodegeneration (Fig. 2b) and decreased MAP2 intensity in 3 × Tg + GFP mice compared to the nonTg + GFP mice, while AAV-NF-α1/CPE significantly increased and AAV-NF-α1/CPE-E342Q partially increased MAP2 intensity in 3 × Tg-AD mice (Fig. 2c).

To evaluate whether AAV-NF-α1/CPE or AAV-NF-α1/CPE-E342Q treatment has any effects on neuroinflammation, GFAP and CD68 immunostaining was performed to assess activation of astrocytes and microglia. Astrocyte activation was comparable across all four groups (Fig. 2d, e), while microglial activation was increased in the 3 × Tg + GFP group compared with the non-Tg mice, but was significantly reduced in 3 × Tg + CPE and 3 × Tg + CPE-E342Q mice compared with the 3 × Tg + GFP mice (Fig. 2f, g). This result indicates that NF-α1/CPE and NF-α1/CPE-E342Q might be involved in the regulation of neuroinflammation via modulating microglial, rather than astrocytic activation.

### AAV-NF-α1/CPE and AAV-NF-α1/CPE-E342Q decrease APP and tau phosphorylation and increase the pro-survival protein Bcl2 in 3 × Tg-AD mice

**Fig. 3:**
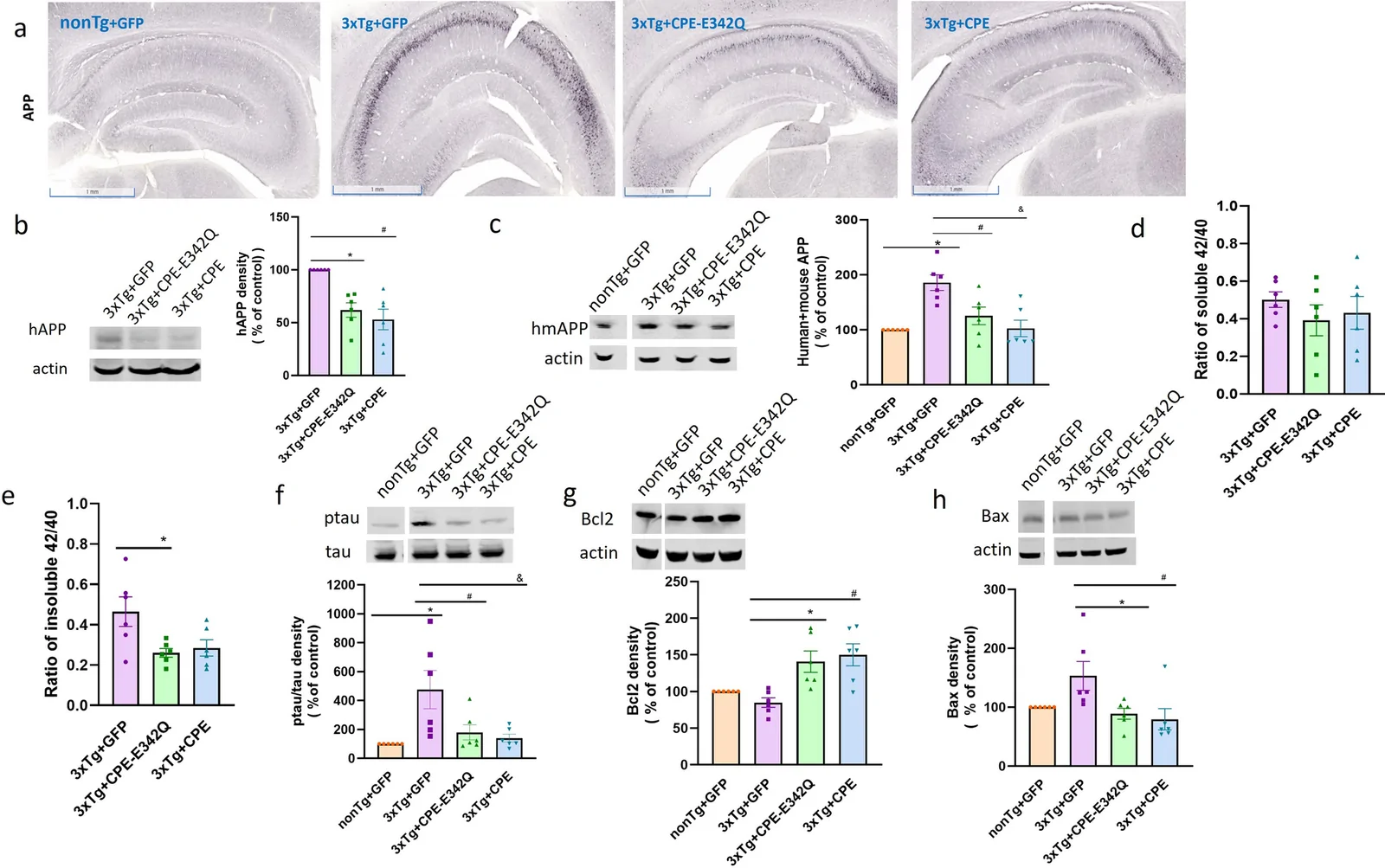
AAV-NF-α1/CPE and AAV-NF-α1/CPE-E342Q decrease APP and tau phosphorylation and increase Bcl2 in 3 × Tg-AD mice. **a** Immunohistochemistry for APP expression after hippocampal stereotaxic injection at the age of 8–9 months. Scale bars, 1 mm. **b** hAPP was decreased by treatment with AAV- NF-α1/CPE or AAV-NF-α1/CPE-E342Q (^*^P = 0.0037, ^#^*P* = 0.0006). One-way ANOVA followed by Tukey’s post-hoc multiple comparison test [*F*(2,15) = 13.16, *P* = 0.0005]. *n* = 6, mean ± SEM. **c** Protein levels of human + mouse APP (hmAPP) were significantly increased in 3 × Tg + GFP compared with nonTg + GFP (* *P* = 0.0008), while treatment with AAV-CPE or AAV-CPE-E342Q decreased hmAPP level in 3 × Tg-AD (^#^*P* = 0.0186; ^&^*P* = 0.0012). One-way ANOVA followed by Tukey’s post-hoc multiple comparison test [*F*(3,20) = 9.301, *P* = 0.0005]. *n* = 6, mean ± SEM. **d** Treatment with AAV-CPE or AAV-E342Q did not induce any significant changes in the ratio of soluble Aβ42/40. *n* = 6, mean ± SEM. **e** Treatment with AAV-CPE-E342Q significantly decreased the ratio of insoluble 42/40 in 3 × Tg-AD (^*^P = 0.0285), and AAV-CPE induced a trend of reduction of the ratio (*P* = 0.0555;). One-way ANOVA followed by Tukey’s post-hoc multiple comparison test [*F*(2,15) = 4.980, *P* = 0.0219]. *n* = 6, mean ± SEM. **f** The ptau/tau ratio was significantly increased in 3 × Tg + GFP compared with nonTg + GFP (^*^P = 0.0078), while treatment with AAV-CPE or AAV-CPE-E342Q decreased the ratio in 3 × Tg-AD (^#^*P* = 0.042; ^&^*P* = 0.0184). One-way ANOVA followed by Tukey’s post-hoc multiple comparison test [*F*(3,20) = 5.555, *P* = 0.0061]. *n* = 6, mean ± SEM. **g** Treatment with AAV-CPE or AAV-CPE-E342Q increased Bcl2 protein level in 3 × Tg-AD (* *P* = 0.0084; ^#^*P* = 0.0021). One-way ANOVA followed by Tukey’s post-hoc multiple comparison test [*F*(3,20) = 8.318, *P* = 0.0009]. *n* = 6, mean ± SEM. **h** Treatment with AAV-CPE and AAV-CPE-E342Q decreased Bax protein level in 3 × Tg-AD (^*^P = 0.0449; ^#^*P* = 0.0184;). One-way ANOVA followed by Tukey’s post-hoc multiple comparison test [*F*(3,20) = 4.274, *P* = 0.0174]. *n* = 6, mean ± SEM

Similar to other AD mouse models, APP immunostaining showed strong intensity in the whole hippocampal area in 3 × Tg-AD mice, while it was very low in the non-Tg mice (Fig. 3a). Some APP immunostaining was also observed in 3 × Tg + CPE and 3 × Tg + E342Q mice (Fig. 3a). Western blotting using an antibody specifically targeting human APP showed that APP was decreased in 3 × Tg mice with both AAV-NF-α1/CPE-E342Q and AAV-NF-α1/CPE treatment (Fig. 3b). Western blotting using an antibody targeting both mouse and human APP showed that the APP protein level was much higher in 3 × Tg + GFP mice compared with the nonTg + GFP mice, and treatment with AAV-NF-α1/CPE-E342Q or AAV-NF-α1/CPE effectively reduced the APP level in 3 × Tg mice (Fig. 3c). In addition, hippocampal AAV-NF-α1/CPE-E342Q or AAV-NF-α1/CPE delivery led to a decreased ratio of insoluble (Fig. 3e), but not soluble Aβ42/40 (Fig. 3d).

Western blotting also revealed a highly increased level of ptau in 3 × Tg + GFP mice compared with the nonTg + GFP mice. However, AAV-NF-α1/CPE-E342Q and AAV-NF-α1/CPE treatment significantly decreased ptau levels in these mice (Fig. 3f).

The protein level of mitochondrial pro-survival protein Bcl2 is downregulated within tangle-bearing neurons of AD patients [29], while expression of Bax, a mitochondrial pro-apoptotic protein, is upregulated in AT8 (a marker for hyperphosphorylated tau)-positive cells in AD patients [30]. Western blotting revealed that Bcl2 and Bax levels were not significantly different between nonTg + GFP and 3 × Tg + GFP, but treatment with AAV-NF-α1/CPE-E342Q or AAV-NF-α1/CPE significantly increased Bcl2 level (Fig. 3g) and decreased Bax level (Fig. 3h), indicating that NF-α1/CPE enhances the Bcl2-mediated pro-survival cascade.

### Proteomic analysis revealed DEPs in the hippocampus of AAV-NF-α1/CPE-treated versus AAV-GFP-treated 3 × Tg-AD mice

**Fig. 4:**
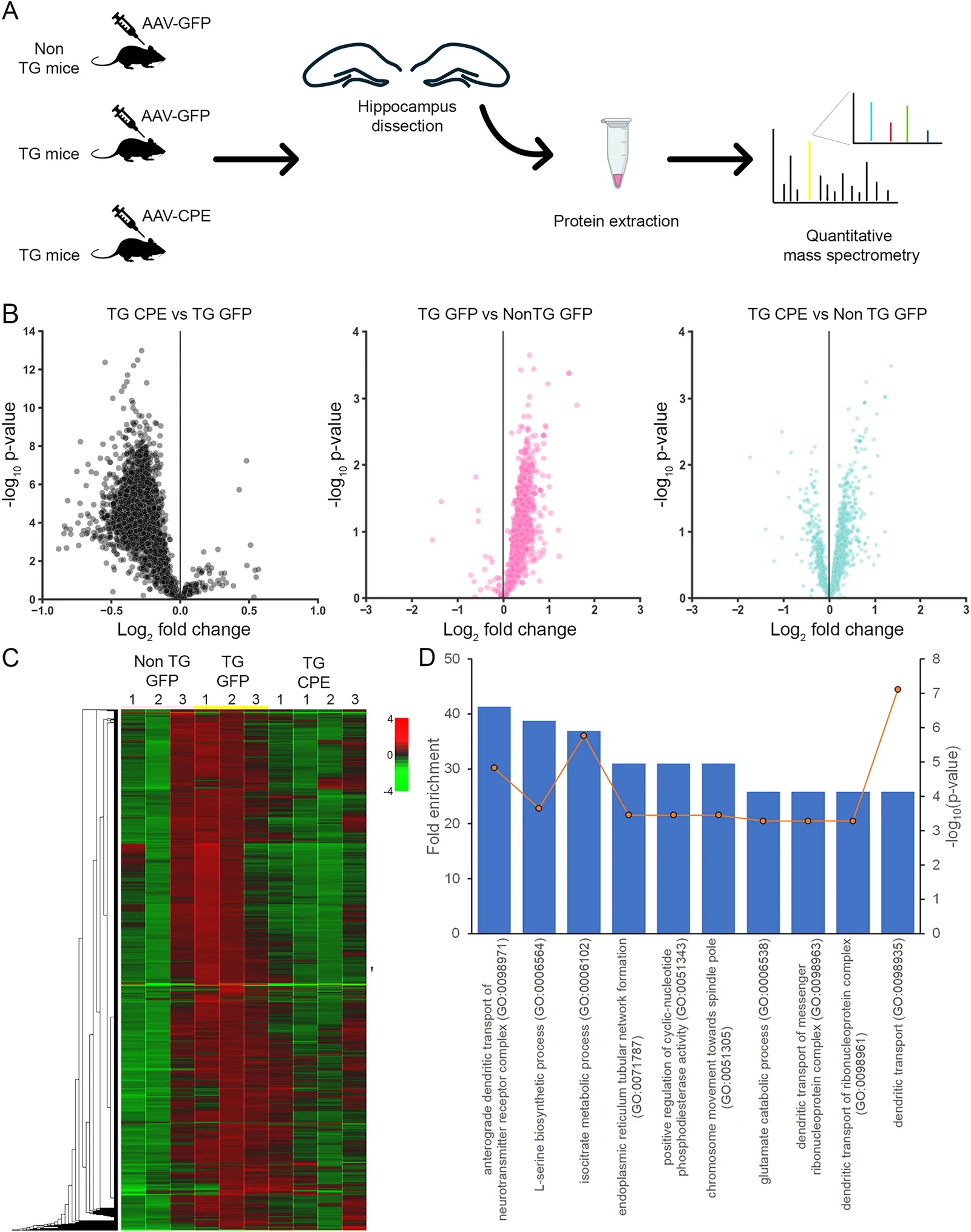
Differentially expressed proteins in hippocampus of AAV-NF-α1/CPE versus AAV-GFP treated 3 × Tg-AD mice. **a** Schematic of quantitative mass spectrometry. Proteins extracted from hippocampus were used for analysis in triplicates. **b** Volcano plots showing quantitative comparison of hippocampal proteins between 3 × Tg + CPE and 3 × Tg + GFP, 3 × TgGFP and nonTg + GFP mice, and 3 × Tg + CPE and nonTg + GFP mice. **c** Protein profile hierarchical heatmap showing protein expression in the hippocampus from 3 groups and their individual replicates (marked as 1, 2 and 3). The replicate 1 was repeated in TG-CPE group as a technical replicate. The cell color represents log_2_ (ratio) to the average abundance across different samples. **d** Top 10 canonical pathways from PANTHER Overrepresentation Test analysis using the dataset of most changed proteins filtered with log_2_ fold change ratio ≥ 0.33 or ≤ -0.33 between 3 × Tg + CPE and 3 × Tg + GFP mice. Blue bars show fold enrichment, and orange circle markers show the significance values plotted as − log (*P*-value). *P*-values were calculated using Fisher exact test with FDR correction

Proteome profiling analysis of hippocampal tissues from nonTg + GFP, 3 × Tg + GFP and 3 × Tg + CPE mice was performed with LC–MS/MS (Fig. 4a). Volcano plots showed that 2814 DEPs were identified (Fig. 4b, Table S3). The volcano plot for comparison of 3 × Tg + GFP versus nonTg + GFP mice showed asymmetric distribution with a majority of proteins showing higher expression in 3 × Tg + GFP (Fig. 4b middle, magenta dots). In comparison, the volcano plot for comparison of 3 × Tg + CPE versus nonTg + GFP mice showed a more symmetric distribution (Fig. 4b right, cyan dots). Notably, there were more DEPs down-regulated than up-regulated in 3 × Tg-AD mice with AAV-NF-α1/CPE treatment (Fig. 4b left panel, grey dots). Comparable proteomic signatures were observed across replicates within the three groups as shown in the protein profile hierarchical heatmap (Fig. 4c). The heatmap data also showed that the general increase in protein expression in the 3 × Tg + GFP mice was reversed in the 3 × Tg + CPE mice. These results suggest that AAV- NF-α1/CPE treatment reversed the global protein level changes associated with AD pathology.

PANTHER Overrepresentation Test was performed to assess whether specific gene sets were represented in the input gene list differently from what is expected by chance. The dataset of most changed proteins were filtered with log_2_ fold change ratio ≥ 0.33 or ≤ − 0.33 in comparison of 3 × Tg + CPE vs 3 × Tg + GFP mice. PANTHER Overrepresentation Test (with Fisher’s Exact test using FDR correction) with the functional category of GO molecular function comparing 3 × Tg + CPE mice vs 3 × Tg + GFP mice showed enrichment in anterograde dendritic transport of neurotransmitter receptor complex, glutamate catabolic process, dendritic transport of messenger ribonucleoprotein complex, dendritic transport of ribonucleoprotein complex, and dendritic transport (Fig. 4d), indicting that AAV-NF-α1/CPE overexpression triggered synaptic and dendritic remodeling.

### Hippocampal delivery of AAV-NF-α1/CPE modulates expression of many AD-associated DEPs in 3 × Tg-AD mice

**Fig. 5:**
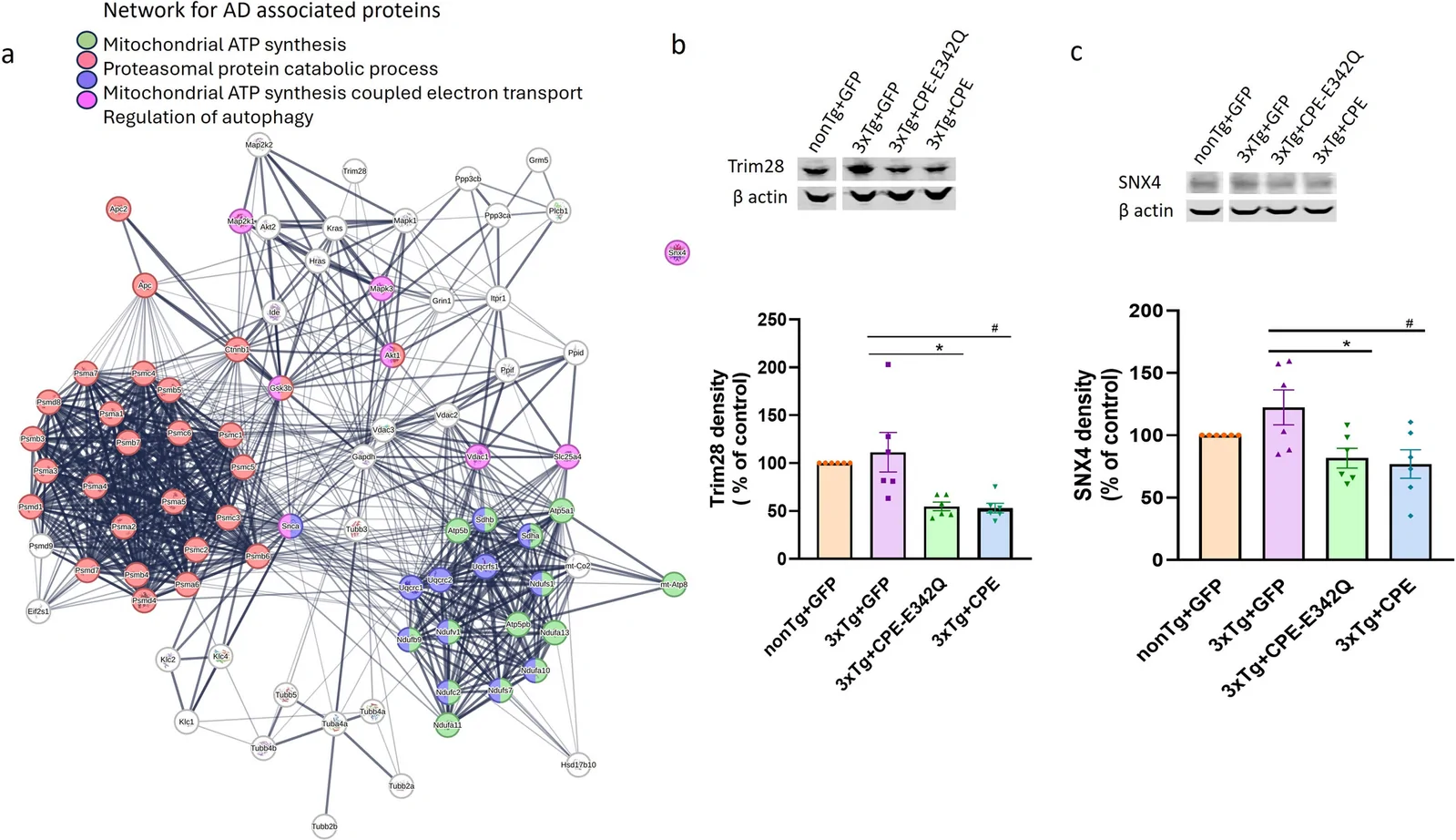
Hippocampal delivery of AAV-NF-α1/CPE modulates expression of AD-associated proteins in 3 × Tg-AD mice. **a** Functional protein network association map generated by String db for proteins annotated to be involved in AD, based on comparison of 3 × Tg + CPE vs 3 × Tg + GFP. **b** Immunoblotting revealed that treatment with AAV-NF-α1/CPE or AAV-CPE-E342Q decreased Trim28 in 3 × Tg-AD (^*^P = 0.0073; ^#^*P* = 0.0057). One-way ANOVA followed by Tukey’s post-hoc multiple comparison test [*F*(3,20) = 7.777, *P* = 0.0012]. *n* = 6 mice per genotype, mean ± SEM. **c** Immunoblotting revealed that treatment with AAV-NF-α1/CPE or AAV-CPE-E342Q decreased Snx4 in 3 × Tg-AD (^*^P = 0.0384; ^#^*P* = 0.0187). One-way ANOVA followed by Tukey’s post-hoc multiple comparison test [*F*(3,20) = 4.399, *P* = 0.0157]. *n* = 6 mice per genotype, mean ± SEM

STRING analysis of dataset comparing 3 × Tg + CPE mice vs 3 × Tg + GFP mice provided Alzheimer disease (KEGG ID: mmu05010) as one of the major KEGG networks, involving 83 proteins (Fig. 5a). Further STRING network analysis of AD-associated proteins in the dataset showed proteins that are involved in mitochondrial ATP synthesis (Fig. 5a, green), proteasomal protein catabolic process (Fig. 5a, red), mitochondrial ATP synthesis electron transport (Fig. 5a, purple), and regulation of autophagy (Fig. 5a, magenta). Among the DEPs associated with AD regulated by NF-α1/CPE in 3 × Tg-AD mice, two new proteins, tripartite motif-containing 28 (Trim28) and sorting nexin-4 (Snx4), were uncovered. Trim28 and Snx4 have been reported to promote tau and Aβ pathology in AD mice, respectively. Quantitative proteomic analysis showed that Trim28 and Snx4 were downregulated with expression of CPE in the hippocampus of 3 × Tg mice. This was further validated by Western blotting, showing that treatment with AAV-NF-α1/CPE or AAV-NF-α1/CPE-E342Q significantly decreased Trim28 and Snx4 levels in 3 × Tg-AD mice compared with 3 × Tg + GFP mice (Fig. 5b, c).

### Hippocampal AAV-NF-α1/CPE delivery modulates synaptic proteins and rescues impaired synaptogenesis in 3 × Tg-AD mice

**Fig. 6:**
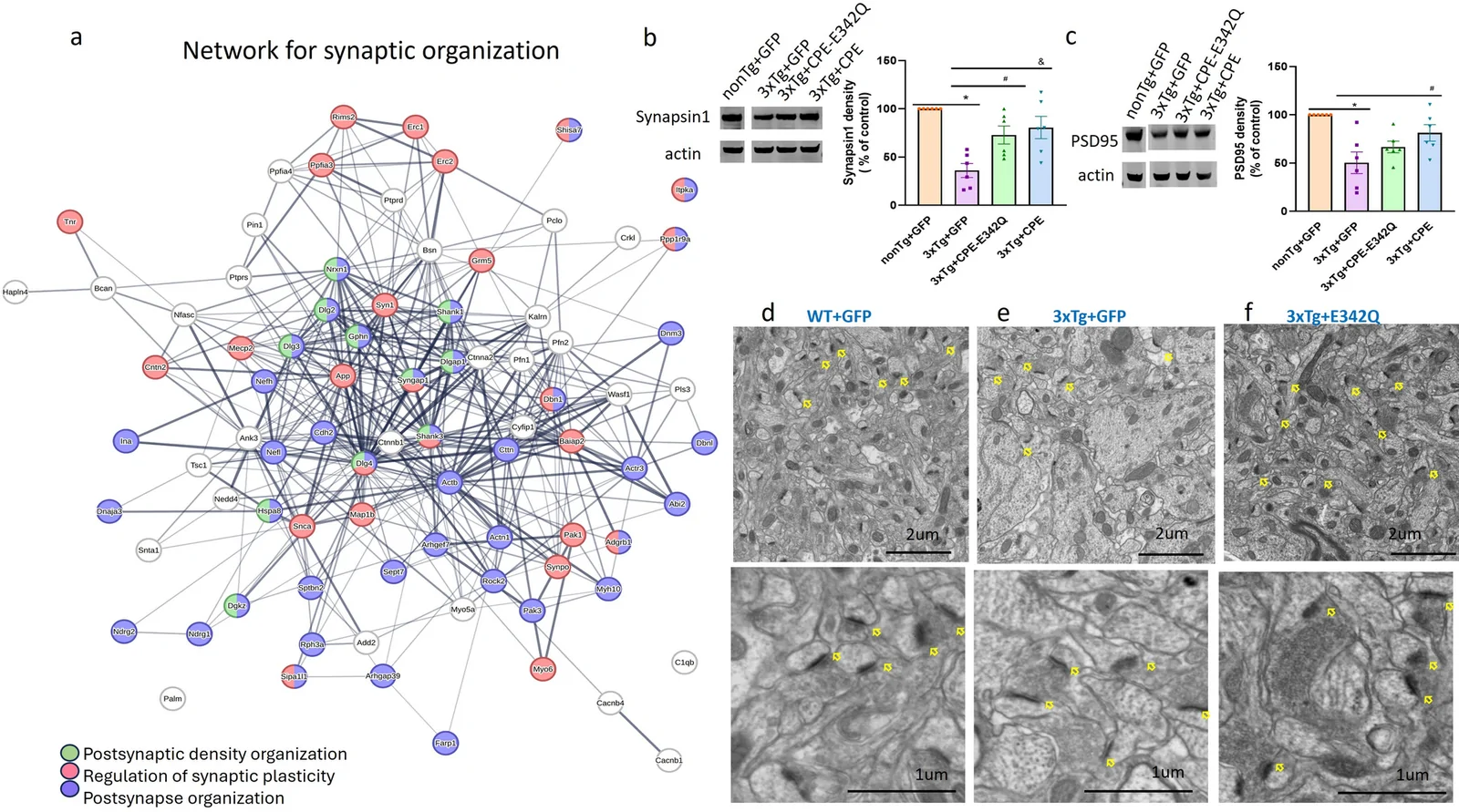
Hippocampal AAV-NF-α1/CPE delivery modulates synaptic proteins and rescues impaired synaptogenesis. **a** Functional protein network association map generated by String db for proteins annotated to be involved in synaptic organization, based on comparison of 3 × Tg + NF-α1/CPE vs 3 × Tg + GFP mice. **b** Synapsin1 protein level was significantly decreased in 3 × Tg + GFP compared with nonTg + GFP (^*^P = 0.0001). Treatment with AAV-NF-α1/CPE or AAV-CPE-E342Q effectively increased synpasin1 in 3 × Tg-AD (^#^*P* = 0.0239; ^&^*P* = 0.0055). One-way ANOVA followed by Tukey’s post-hoc multiple comparison test [*F*(3,20) = 10.52, *P* = 0.0002]. *n* = 6 mice per genotype, mean ± SEM. **c** Synaptic marker PSD95 was significantly decreased in 3 × Tg + GFP compared with nonTg + GFP (^*^P = 0.001). Treatment with AAV-NF-α1/CPE increased PSD95 in 3 × Tg-AD (^#^*P* = 0.0454). One-way ANOVA followed by Tukey’s post-hoc multiple comparison test [*F*(3,20) = 7.573, *P* = 0.0014]. *n* = 6 mice per genotype, mean ± SEM. **d–f** Electron microscopic images of nonTg + GFP (**d**), 3 × Tg + GFP (**e**) and 3 × Tg + CPE-E342Q (**f**). Arrows indicate synapses. scale bars, 2 μm in upper panels, 1 μm in bottom panels

Synaptic loss is highly correlated with cognitive impairments in AD patients [31–33]. Consistently, decreased synaptic density and activity have also been observed in AD mouse models [34, 35]. STRING pathway analysis of dataset comparing expression between 3 × Tg + CPE mice versus 3 × Tg + GFP mice revealed that AAV-NF-α1/CPE treatment modulates expression of multiple proteins involved in synaptogenesis in 3 × Tg-AD mice (Fig. 6a). Further analysis of synaptic organization network revealed that the proteins are primarily involved in postsynaptic density organization (Fig. 6a, green), regulation of synaptic plasticity (Fig. 6a, red), and postsynaptic organization (Fig. 6a, purple).

To test the hypothesis provided by our proteomic analysis, we investigated two major synaptic proteins Synapsin1 and postsynaptic density protein 95 (PSD95). Synapsin1 is mainly distributed in the vesicles of presynaptic terminals and regulates neurotransmitter release [36–38]. PSD95 is a critical component of post-synaptic density that interacts with many post-synaptic proteins. Western blotting analysis showed significant decreases of Synapsin1 and PSD95 protein levels in the 3 × Tg + GFP mice compared with the nonTg + GFP mice (Fig. 6b, c). Treatment with AAV-NF-α1/CPE significantly upregulated expression of Synapsin1 (Fig. 6b) and PSD95 (Fig. 6c) in 3 × Tg-AD mice. AAV-NF-α1/CPE-E342Q treatment increased Synapsin1 protein level and induced a trend of increase of PSD95 in 3 × Tg-AD mice comparison with the 3 × Tg + GFP mice (Fig. 6c). Electron microscopy showed post-synaptic thickening in synapses in the hippocampus of nonTg + GFP (Fig. 6d) and 3 × Tg + CPE-E342Q (Fig. 6f) mice, in contrast to the thin post-synaptic structures in the 3 × Tg + GFP mice (Fig. 6e). This finding suggested that defective synaptogenesis in the 3 × Tg-AD mice is reversed with AAV-NF-α1/CPE or AAV- NF-α1/CPE-E342Q treatment.

### AAV-NF-α1/CPE and AAV-NF-α1/CPE-E342Q treatment restore autophagic activity in 3 × Tg-AD mice

**Fig. 7:**
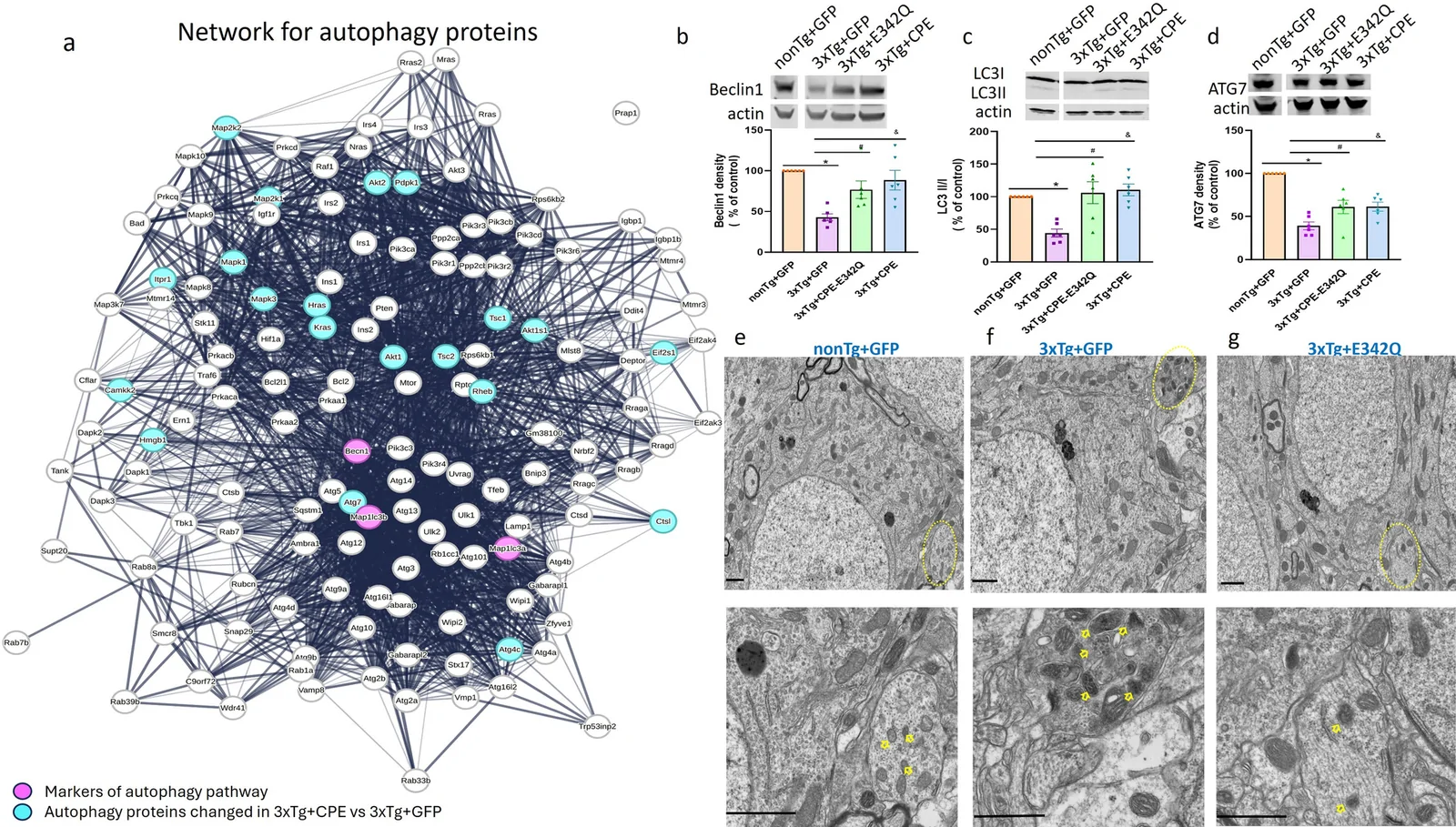
AAV-NF-α1/CPE and AAV-NF-α1/CPE-E342Q treatment restore autophagic activity in 3 × Tg-AD mice. **a** The String db functional protein network association map of autophagy-related proteins. The proteins highlighted in cyan were detected in our experimental dataset comparing 3 × Tg + CPE vs 3 × Tg + GFP. Beclin1 and Map1lc3a/b (LC3 II/I) labeled in magenta are prominent markers of autophagy pathway. **b** Western blotting showing that Beclin1 was significantly decreased in 3 × Tg + GFP compared with nonTg + GFP (^*^P = 0.0005). Treatment with AAV-NF-α1/CPE and CPE-E342Q both increased Beclin1 in 3 × Tg-AD (^#^*P* = 0.0426; ^&^*P* = 0.0049). One-way ANOVA followed by Tukey’s post-hoc multiple comparison test [*F*(3,20) = 8.743, *P* = 0.0007]. *n* = 6 mice per genotype, mean ± SEM. **c** Western blotting showing that the LC3II/LC3I ratio was significantly decreased in 3 × Tg + GFP compared with nonTg + GFP (^*^P = 0.0041). Treatment with AAV-NF-α1/CPE and CPE-E342Q both increased the ratio in 3 × Tg-AD (^#^*P* = 0.0015; ^&^*P* = 0.0008). One-way ANOVA followed by Tukey’s post-hoc multiple comparison test [*F*(3,20) = 9.579, *P* = 0.0004]. *n* = 6 mice per genotype, mean ± SEM. **d** Western blotting showing that ATG7 was significantly decreased in 3 × Tg + GFP compared with nonTg + GFP (*P < 0.0001). Treatment with AAV-NF-α1/CPE and CPE-E342Q both increased ATG7 in 3 × Tg-AD mice (^#^*P* = 0.0347; ^&^*P* = 0.0309). One-way ANOVA followed by Tukey’s post-hoc multiple comparison test [*F*(3,20) = 23.71, *P* < 0.0001]. *n* = 6 mice per genotype, mean ± SEM. **e, f, g** Electron microscopic images of nonTg + GFP (**e**), 3 × Tg + GFP (**f**) and 3 × Tg + E342Q (**g**). Dashed circles: neurites (defined by the structure of synapses) containing autophagosomes. Arrowheads indicate autophagosomes containing dense core materials. Scale bars, 1 μm

Autophagy plays a crucial role in the pathogenesis of neurodegeneration [39]. Post-mortem studies have reported accumulation of autophagosomes containing large amounts of dense core materials in the dystrophic neurites around amyloid plaques in the cortex of AD patients, linking impaired autophagy with AD [40]. Our proteomic analysis revealed a network of DEPs that are associated with autophagy pathways (Fig. 7a). STRING analysis of dataset comparing 3 × Tg + CPE mice versus 3 × Tg + GFP mice revealed alterations of 135 autophagy-associated proteins.

To examine the autophagy-related proteins that may be modulated by CPE or CPE-E342Q to rescue impaired autophagy in 3 × Tg-AD mice, immunoblotting and electron microscopy studies were performed. Beclin1 and Map1lc3a/b (LC3 II/I) are established autophagy markers. Beclin1 facilitates the assembly of autophagosomes via interacting with other proteins such as PI3KC3 (class III type phosphoinositide 3-kinase)/Vps34 [41, 42]. LC3II is a modified form of microtubule-associated light chain 3 (LC3 I) protein which is recruited to autophagosome membrane and later degraded by lysosomal hydrolases [43]. Western blots showed that Beclin1 was significantly decreased in 3 × Tg + GFP mice compared with nonTG + GFP mice, while treatment with AAV-NF-α1/CPE or AAV-NF-α1/CPE-E342Q increased the level of Beclin1 compared to the 3 × Tg + GFP group (Fig. 7b). In addition, the LC3II/LC3I ratio was decreased in the 3 × Tg-AD mice compared to the non-Tg mice, and increased with AAV-NF-α1/CPE or AAV-NF-α1/CPE-E342Q treatment (Fig. 7c). The network analysis revealed alterations of the autophagy pathway protein ATG7 downstream of Map1lc3a/b (LC3 II/I). Hence we examined regulation of its expression by NF-α1/CPE. Western blotting revealed that the ATG7 level was decreased in 3 × Tg-AD mice, but increased with AAV-NF-α1/CPE or AAV-NF-α1/CPE-E342Q treatment (Fig. 7d).

Electron microscopy showed that autophagosomes in normal neurites in the hippocampus of nonTg + GFP mice had little accumulation of dense material (Fig. 7e); in contrast, autophagosomes in the 3 × Tg + GFP mice contained highly dense undigested materials in dystrophic neurites (Fig. 7f). In the 3 × Tg + CPE-E342Q mice, autophagosomes in neurites contained less accumulation of materials compared to 3 × Tg + GFP (Fig. 7g). These results indicate that AAV-NF-α1/CPE and AAV-NF-α1/CPE-E342Q treatment rescues impaired autophagy in 3 × Tg-AD mice by up-regulating expression of critical proteins Beclin1, LC3 and ATG7.

## Discussion

**Fig. 8:**
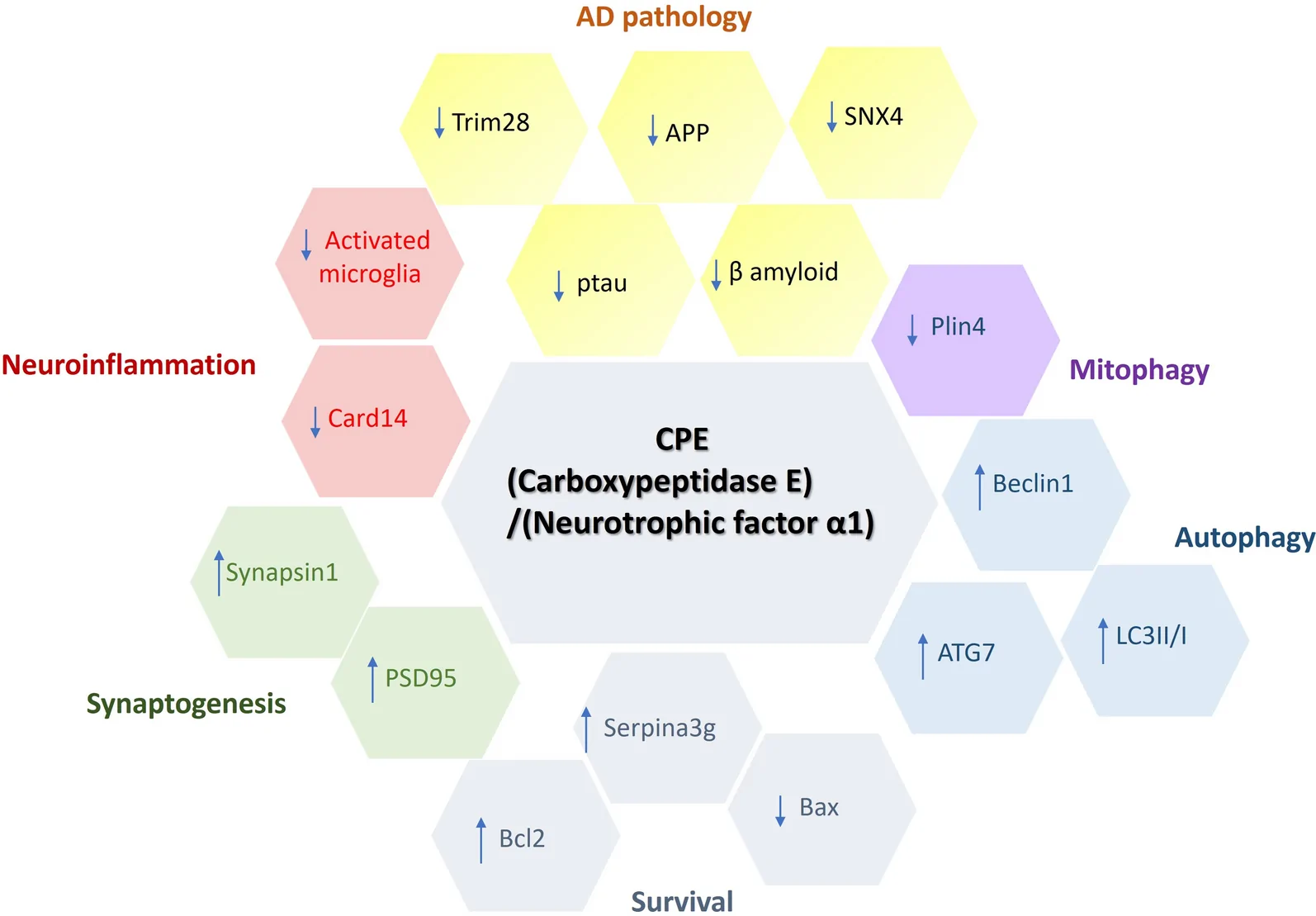
Summary of proteins verified to be regulated by NF-α1/CPE in 3 × Tg-AD mice. NF-α1/CPE downregulates characteristic pathological proteins including APP, β amyloid and ptau. Additionally, Trim28 and Snx4 which are involved in the production of pathological proteins, are reduced by NF-α1/CPE treatment. AD-associated neuroinflammation, especially increased activation of microglia, is reduced by NF-α1/CPE treatment as evidenced by reduced number of activated microglia and decreased level of pro-inflammatory protein Card14. Autophagy markers Beclin1, ATG7 and ratio of LC3II/I, are all increased by NF-α1/CPE treatment, indicating higher activity of autophagy. Synaptogenesis markers such as Synapsin1 and PSD95 are increased by NF-α1/CPE, suggesting increased synaptogenesis. NF-α1/CPE also acts as a neuroprotector by upregulating survival proteins. For instance, mitochondria survival protein Bcl2 is enhanced, and pro-apoptotic protein Bax is reduced by NF-α1. Serpina3g, a protein that upregulates survival, is also increased by NF-α1/CPE. Plin4, an inhibitor of mitophagy, is reduced by NF-α1/CPE

Recent work from several laboratories have demonstrated that delivery of AAV- or lentivirus-NF-α1/CPE or upregulation of NF-α1/CPE expression by agomirs can rescue AD pathology and ameliorate cognitive decline in three different AD mouse models [13, 44, 45]. In the study by Fan et al. [44], conditional knockout of NF-α1/CPE in 5 × FAD mice across different ages intensified AD pathology. Furthermore, a direct dose-dependent correlation between CPE overexpression and the decrease of amyloid plaque formation in the hippocampus was found [44]. In the study by Jiang et al., two agomirs administered through nasal instillation or via cerebral ventricular injection increased CPE expression, which subsequenly enhanced neurogenesis in the dentate gyrus to reverse AD pathology and cognitive dysfunction. The proposed mechanism is the increased CPE expression after agomir treatment, which enhances BDNF (brain-derived neurotrophic factor) and FGF2 (fibroblast growth factor 2) levels known to promote neurogenesis [45]. Our previous study has shown that hippocampal delivery of AAV-NF-α1/CPE into 3 × Tg-AD mice increases Bcl2 and decreases Bax, a mechanism known to mediate neuroprotection [13]. Additionally, Serpina3g (Serine protease inhibitor A3G), a pro-survival protein, is up-regulated by AAV-NF-α1/CPE treatment [13]. Moreover, the AAV-NF-α1/CPE-treated 3 × Tg-AD mice demonstrate suppression of APP expression through decreasing the transcription factors Sp1 and Hsf1, which bind the promoter of *APP* to regulate its expression. Levels of the pro-inflammatory protein Card14 and the mitophagy inhibitor Plin4 were downregulated by AAV-NF-α1/CPE treatment, mitigating neuroinflammation and promoting mitophagy, respectively. These previous findings have propelled NF-α1/CPE as a potentially important therapeutic agent for treatment of AD (Fig. 8).

In the current study, we showed that human CPE, which is 96.6% identical to mouse CPE in amino acid sequence, has no functional difference from mouse CPE [13] in neuroprotection and reversal of AD pathology and cognitive dysfunction in AD mice.

The results of proteomic analysis uncovered novel proteins involved in AD pathogenesis and further illuminated the mechanisms underlying the actions of NF-α1/CPE in rescuing AD pathology and cognitive dysfunction in 3 × Tg-AD mice. These include restoring protein levels of Synapsin1 and PSD95 that are involved in synaptogenesis (Fig. 8). Analysis of DEPs in the autophagy pathway network revealed that NF-α1/CPE restored levels of Beclin1 and Map1lc3a/b (LC3II/I), and up-regulated the expression of its downstream protein ATG7, which were confirmed by Western blotting. ATG7 is a crucial protein in the autophagy pathway, acting as an E1-like enzyme that initiates the process of autophagy and plays a vital role in the formation and extending of autophagosomes that engulf cellular waste and damaged components for recycling [46] (Fig. 8). Furthermore, Trim28 and Snx4, which showed aberrant levels in AD mice, were decreased with NF-α1/CPE treatment. Trim28 plays a critical role in regulating α-Syn and tau levels. Reduction of Trim 28 leads to decreased tau and α-Syn levels in *Drosophila* [47] and mice [48]. Snx4 regulates Aβ production. Reduction of Snx4 activity leads to decreased steady-state level of BACE1 and subsequent attenuation of Aβ production [49]. Thus, NF-α1/CPE acts in a multifaceted manner to restore cognitive dysfuntion and ameliorate pathology in AD mice.

Numerous proteomic studies using animal models and in AD patients have indicated that AD is a very complex disease, exhibiting dysregulation of multiple regulatory metabolic pathways [50–52]. An analysis of proteomic studies involving 38 reports in clinical patients with AD revealed that synaptic activity- and vesicle-associated proteins are altered in early stage of AD, and mitochondria function-associated proteins are changed in more advanced stage of AD [53]. Aberrant autophagy has also been reported in AD patients [54]. Indeed, findings from our proteomic study in 3 × Tg mice mirror those from clinical studies. Dysregulation of vast numbers of proteins associated with mitochondrial activity, proteasomal protein catabolic process, dendritic transport and glutamate catabolic process and autophagy were also identified in our AD mice. Given the mitochondrial and autophagic signatures revealed by proteomics analysis, in the future we will continue to investigate the role of NF-α1/CPE in mitochondrial function and ATP production, as well as the significance of TFEB (transcription factor EB) in mitigating autophagy dysfunction.

Although there were no exact common dysregulated proteins identified in our 3 × Tg proteomic study with those from clinical AD proteomic analysis published thus far, there were many dysregulated proteins with common functions. For example, glutamate ionotropic receptor AMPA type subunit 2 (GRIA2) has been found decreased in AD patients [53], and Gria1 was also altered in the 3 × Tg mice with NF-α1/CPE treatment (Fig. S1), suggesting the role of AMPA subunits in the pathogenesis of AD. Changes of HSPB1 (heat shock protein beta 1) in AD brains have been reported [53, 55] and alteration of Hspa1a (Heat Shock Protein Family A (Hsp70) Member 1A) was observed in the 3 × Tg mice with NF-α1/CPE treatment (Fig. S1). These findings indicate the involvement of HSP families in the progression of AD. Hence, our proteomic study uncovered new insights into proteins that are altered in AD pathology and progression, providing an avenue for further translational and clinical studies, such as developing biomarkers for early detection of AD and monitoring treatment efficacy.

## Conclusions

AD is a multifactorial disease, yet at present most therapeutic approaches have focused on single targets such as elimination of tau and amyloid accumulation in the brain, with only a small amount of success for long-term reversal of cognitive dysfunction [56–59]. Moreover, these drugs produce many side effects [60]. Our current study together with previous work has strengthened the evidence that NF-α1/CPE is an excellent candidate therapeutic agent for treating AD and potentially other neurodegenerative diseases. First, we have demonstrated that NF-α1/CPE is different from other therapeutics in its ability to normalize and modulate expression of proteins involved in numerous cell biological processes, metabolic pathways, synaptogenesis and autophagy in neurons and glial cells to regain homeostasis. Second, our proteomic study uncovered new proteins linked to AD and provided insights into the mechanism of actions of NF-α1/CPE in rescuing AD pathology. Finally, we have previously demonstrated that overexpression of NF-α1/CPE via the AAV-gene therapy in normal mice has no apparent side effects on proteins disrupted in AD or cognitive function [13]. While our studies were conducted in male 3 × Tg-AD mice, other studies using both male and female, or female AD mouse models [44] have also shown the ability of NF-α1/CPE to reverse AD pathology and cognitive dysfunction, suggesting no gender distinction in the efficacy of this treatment approach.

## Supplementary Information

Additional file 1. **Fig. S1** List of all proteins detected by quantitative mass spectrometry. **Table S1.** List of antibodies for western blot. **Table S2**. List of antibodies for immunohistochemistry

Additional file 2. **Table S3.** DEPs in the hippocampus of AAV-NF-α1/CPE-treated versus AAV-GFP-treated 3×Tg-AD mice.

Additional file 3. Original uncropped Western blots.

## Data Availability

All data sets generated during and/or analyzed during the current study are available from the corresponding authors on reasonable request.

## Abbreviations

AD: Alzheimer’s disease
APP: Amyloid precursor protein
ATG7: Autophagy related 7
Gria: Glutamate ionotropic receptor AMPA type subunits
LC3: Microtubule-associated protein 1A/1B-light chain 3
NF-α1/CPE: Neurotrophic Factor-α1/carboxypeptidase E
Plin4: Perilipin 4
PSD95: Postsynaptic density protein 95
Serpina3g: Serine protease inhibitor A3G
Snx4: Sorting nexin 4
Trim28: Tripartite motif containing 28
