# Correction: Neurotrophic factor-α1/carboxypeptidase E regulates critical protein networks to rescue neurodegeneration, defective synaptogenesis and impaired autophagy in Alzheimer’s disease mice

**DOI:** 10.1186/s40035-026-00574-0

**Published:** 2026-08-25

**Authors:** Lan Xiao, Pranav Sharma, Xuyu Yang, Daniel Abebe, Y. Peng Loh

**Affiliations:** 1 Section On Cellular Neurobiology, Eunice Kennedy Shriver National Institute of Child Health and Human Development, National Institutes of Health, 49, Convent Drive, Bldg. 49, Rm 6A-10, NICHD, NIH, Bethesda, MD 20892 USA; 2 Xosomix LLC, 3210 Merryfield Row, San Diego, CA 92121 USA

**Correction: Translational Neurodegeneration (2025) 14:59** 10.1186/s40035-025-00520-6

Following the publication of original article [1], the authors reported that there is an error in the lead sentence of Fig. 1’s legend, which should be corrected from:

AAV-NF-α1/CPE and AAV-NF-α1/CPE-E342Q decrease APP and tau phosphorylation and increase Bcl2 in 3 × Tg-AD mice.

To:

AAV-NF-α1/CPE and AAV-NF-α1/CPE-E342Q rescue memory deficits in 3 × Tg-AD mice.

The original article [1] has been corrected.
