# Supplementary material for: Neurotrophic factor-α1/carboxypeptidase E regulates critical protein networks to rescue neurodegeneration, defective synaptogenesis and impaired autophagy in Alzheimer’s disease mice
Source: Transl Neurodegener. 2025 Nov 26;14:59. doi: 10.1186/s40035-025-00520-6 (PMC12648813; doi:10.1186/s40035-025-00520-6)
Supplement: Supplementary file 1 — Additional file 1. Fig. S1 List of all proteins detected by quantitative mass spectrometry. Table S1. List of antibodies for western blot. Table S2. List of antibodies for immunohistochemistry [file 40035_2025_520_MOESM1_ESM.docx]

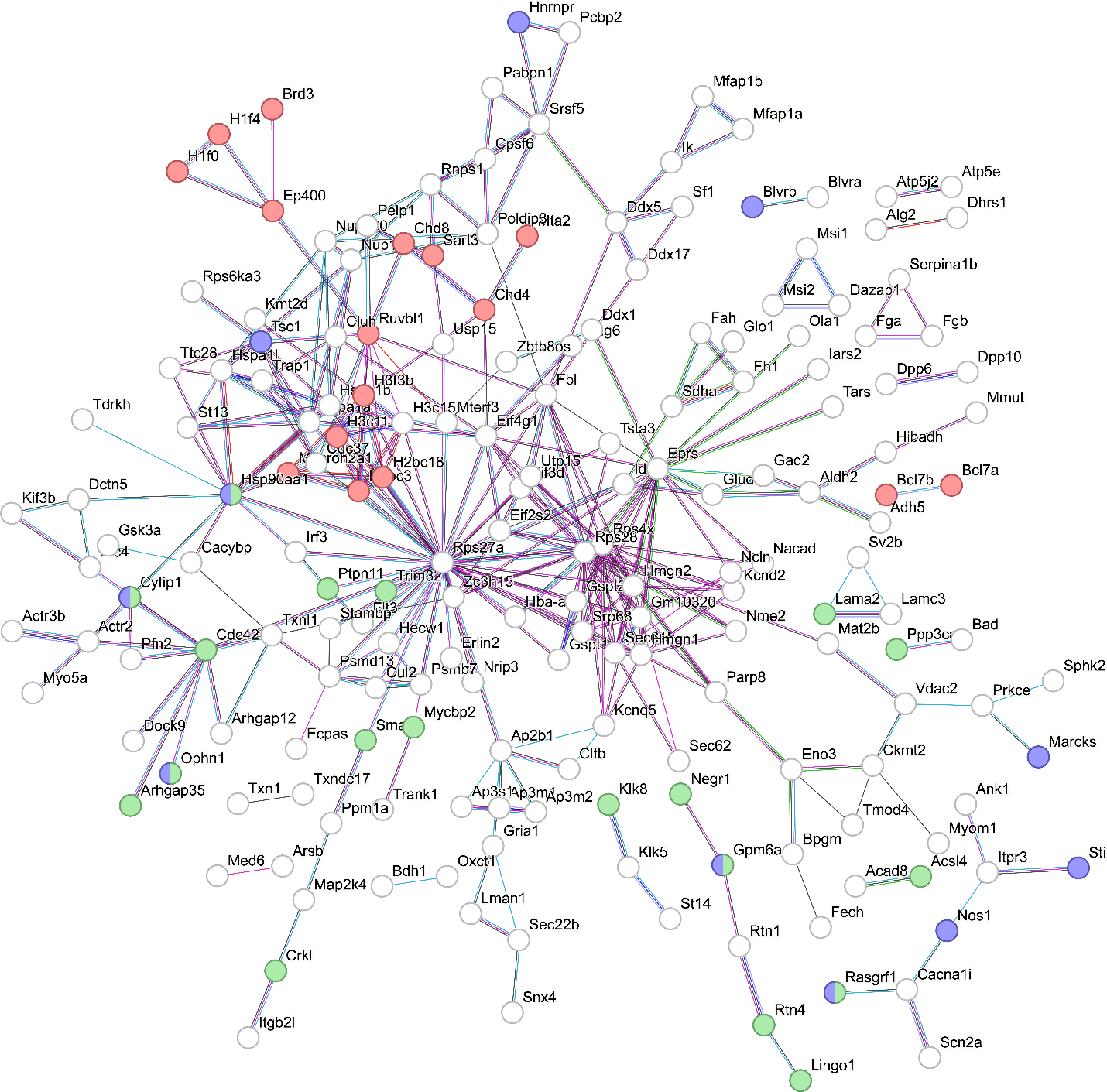


**Fig. S1**

List of all proteins detected by quantitative mass spectrometry

The dataset of proteins with at least 3 peptides detected on mass spectrometry and >1.25X change between TG mice injected with CPE vs TG mice injected with GFP was analyzed for Functional Enrichment Analysis using “Proteins with Values/Ranks” analysis on STRING database (https://string-db.org). One of the enriched functional signaling network shows proteins involved in nucleosome assembly (red), central nervous system development (green) and axon development (blue).

**Table S1.** List of antibodies for western blot

| Antibody | Host | Dilution | Catalog number | Source |
| --- | --- | --- | --- | --- |
| CPE | Mouse | 1:3000 | 610759 | BD Biosciences |
| β-actin | Rabbit | 1:3000 | mAb #4970s | Cell signaling |
| β-actin | Mouse | 1:1000 | A5316 | Sigma Aldrich |
| Trim28(KAP1) | Rabbit | 1:2000 | ab10484 | Abcam |
| SNX4(G3) | Mouse | 1:1000 | sc-271403 | Santa Cruz |
| ATG7 | Rabbit | 1:1000 | 10088-2-AP | Proteintech |
| ptau | Mouse | 1:2000 | sc-32275 | Santa Cruz |
| tau | Mouse | 1:2000 | sc-32274 | Santa Cruz |
| hmAPP | Rabbit | 1:500 | ab32136 | Abcam |
| hAPP | mouse | 1:2000 | 13-0200 | Invitrogen |
| Synapsin1 | Rabbit | 1:2000 | ab64581 | Abcam |
| PSD95 | Rabbit | 1:2000 | ab18258 | Abcam |
| Bcl2 | Mouse | 1:2000 | 15071 | Cell signaling |
| Bax | Rabbit | 1:2000 | #2772 | Cell signaling |
| Beclin1(E8) | Mouse | 1:2000 | sc-48341 | Santa Cruz |
| LC3 | Rabbit | 1:1000 | 14600-1-AP | Proteintech |

**Table S2.** List of antibodies for immunohistochemistry

| Antibody | Host | Dilution | Catalog number | Source |
| --- | --- | --- | --- | --- |
| GFAP | Rabbit | 1:1500 | z0334 | Dako |
| CD68 | Rabbit | 1:15000 | 600-401-R10 | Rockland |
| APP | Rabbit | 1:3000 | 51-2700 | ThermoScientific |
| MAP 2 | Chicken | 1:1500 | cpca-map2 | EnCor |
| Anti-Rabbit Cy3 | Donkey | 1:500 | 711-165-152 | Jackson Lab |
| Anti-Chicken Cy3 | Donkey | 1:500 | 703-165-155 | Jackson Lab |
| Anti-Rabbit Biotinylated | Goat | 1:1000 | ba-1000 | Vector |
