# Supplementary material for: Neurotrophic factor-α1/carboxypeptidase E regulates critical protein networks to rescue neurodegeneration, defective synaptogenesis and impaired autophagy in Alzheimer’s disease mice
Source: Transl Neurodegener. 2025 Nov 26;14:59. doi: 10.1186/s40035-025-00520-6 (PMC12648813; doi:10.1186/s40035-025-00520-6)
Supplement: Supplementary file 3 — Additional file 3. Original uncropped Western blots. [file 40035_2025_520_MOESM3_ESM.docx]

| **lane label:** | |  |
| --- | --- | --- |
| **1** | **nonTg+GFP** | |
| **2** | **nonTg+E342Q** | |
| **3** | **3xTg+GFP** |  |
| **4** | **3xTg+E342Q** | |
| **5** | **3xTg+CPE** | |
